# Comprehensive analysis of phosducin-like 3 as a diagnostic, prognostic and immunological marker in pan-cancer

**DOI:** 10.3389/fimmu.2025.1604179

**Published:** 2025-07-10

**Authors:** Zihao Li, Jiayi Li, Fengchang Li, Honghua Liang, Zuotao Wu, Yongjie Zhu, Jusen Nong, Ting Zhuo, Peng Luo, Lingyun He, Weijia Huang, Jianbin Cao

**Affiliations:** ^1^ Department of Thoracic Surgery, Liuzhou People’s Hospital affiliated to Guangxi Medical University, Liuzhou, Guangxi, China; ^2^ Department of Nephrology, Liuzhou People’s Hospital affiliated to Guangxi Medical University, Liuzhou, Guangxi, China; ^3^ Department of Endocrinology, The First Affiliated Hospital of Guangxi Medical University, Nanning, Guangxi, China; ^4^ Department of Cardio-Thoracic Surgery, The First Affiliated Hospital of Guangxi Medical University, Nanning, Guangxi, China; ^5^ Department of Pediatric Surgery, The First Affiliated Hospital of Guangxi Medical University, Nanning, Guangxi, China; ^6^ Department of Respiratory Medicine, The First Affiliated Hospital of Guangxi Medical University, Nanning, Guangxi, China

**Keywords:** PDCL3, pan-cancer, biomarker, bioinformatics analysis, immune infiltration

## Abstract

**Background:**

Phosducin-like 3 (PDCL3), a member of the photoreceptor family, is involved in angiogenesis and apoptosis. However, there is no pan-cancer analysis, and few studies have explored the effect of PDCL3 on tumor immune infiltration.

**Method:**

Public datasets were used to explore the diagnostic and prognostic value of PDCL3. The relationship between PDCL3 expression and immune infiltration, tumor mutation burden (TMB), and microsatellite instability (MSI) was investigated. Additionally, the therapeutic value of PDCL3 was explored. Finally, differences in PDCL3 expression across cell clusters were analyzed using single-cell datasets. *In vitro* cellular assays were performed to assess the impact of PDCL3 expression on the proliferative capacity, migratory potential, and invasive properties of non-small cell lung cancer (NSCLC) cells.

**Results:**

PDCL3 expression was upregulated in most tumors and correlated with poor outcomes, showing diagnostic and prognostic value. In addition, PDCL3 expression exhibited a positive correlation with infiltration of T helper 2 (Th2) cells and a negative correlation with infiltration of plasmacytoid dendritic cells (pDCs) across a variety of tumors. A relationship was also found between PDCL3 expression and TMB and MSI. Single-cell dataset analysis confirmed that PDCL3 expression was primarily in cancer cells and macrophages. *In vitro* functional analyses demonstrated that genetic silencing of PDCL3 significantly reduced proliferative rates, migratory activity, and invasive potential in pulmonary carcinoma cell models.

**Conclusions:**

PDCL3 may contribute to cancer progression and is a potential candidate biomarker for pan-cancer diagnosis and prognosis. These findings suggest that targeting PDCL3 may provide a valuable strategy for cancer immunotherapy.

## 1 Introduction

The incidence of cancer has gradually increased, primarily due to genetic and environmental influences, and it has become a pivotal factor affecting people’s quality of life and life expectancy (1, 2). In recent years, the application and promotion of immunotherapy and targeted therapy have improved the overall prognosis for cancer patients. However, the overall effect remains unsatisfactory due to the development of acquired drug resistance (3–5). Therefore, identifying tumor markers for diagnosis, prognosis, and treatment, and not only focusing on individual tumors but also conducting a comprehensive pan-cancer analysis, can help clarify the pathogenesis of tumors and provide new therapeutic insights.

Phosducin-like 3 (PDCL3), a member of the Phosducin-like protein (PhLP) family, is also known as Phosducin-like Protein 2A (PhLP2A) (6, 7). As a chaperone protein, PDCL3 is involved in the regulation of vascular endothelial growth factor receptor 2 (VEGFR-2) expression, promoting angiogenesis (8). Angiogenesis, the development of new blood vessels and the formation of a mature blood vessel network based on existing capillaries, has been confirmed as one of the key drivers of the progression of various malignant tumors (9). Anti-angiogenesis is a promising approach in cancer treatment. Therefore, it is speculated that PDCL3 plays a key role in both cancer progression and treatment.

This study comprehensively investigated PDCL3 in pan-cancer, including an in-depth analysis of its expression differences, diagnostic and prognostic value, immune infiltration, and potential implications for immunotherapy, aiming to clarify the clinical effect of PDCL3 on cancer.

## 2 Materials and methods

### 2.1 Data acquisition

The Cancer Genome Atlas (TCGA) datasets, which include prognostic data, clinical information, and gene expression profiles, were obtained from UCSC Xena (xenabrowser.net). These datasets include gene expression profiles from 34 types of cancer, covering tumor tissues (N=9807) and normal tissues (N=8295). Gene expression data were transformed using log2(x+1). Single-cell sequencing datasets were downloaded from the Gene Expression Omnibus (GEO) (https://www.ncbi.nlm.nih.gov/geo/), including GSE189357, GSE242889, GSE263995, and GSE184198.

### 2.2 Differential analysis of PDCL3

RNA expression differences of PDCL3 between multiple human tumor cases and corresponding non-tumor cases were analyzed and compared using the TCGA dataset. The differential expression of PDCL3 was further validated at the protein level using UALCAN (https://ualcan.path.uab.edu/) and the Human Protein Atlas (HPA) (http://www.proteinatlas.org/). Furthermore, RNA expression of PDCL3 was compared across tumor samples at different stages.

### 2.3 Analysis of the diagnostic efficacy of PDCL3 for pan-cancer

The expression level of PDCL3 in various tumors was used to differentiate tumor samples from normal samples. The R package ROCR was applied to generate the receiver operating characteristic (ROC) curve to evaluate the diagnostic efficacy of PDCL3.

### 2.4 Survival analysis of PDCL3

The Kaplan–Meier (KM) method was employed to construct survival curves based on PDCL3 expression. The R packages survminer and survival were used to perform curve plotting. The optimal cut-off value was determined to divide tumor cases into two groups for survival analysis.

### 2.5 Mutational characterization of PDCL3 in tumors

The cBioPortal database (http://www.cbioportal.org/) was used to examine the mutation characteristics of PDCL3 across pan-cancers.

### 2.6 Analysis of the correlation between PDCL3 and immune infiltration in various tumors

The R package GSVA was used to quantify the level of immune infiltration in tumor cases, followed by an analysis of the correlation between PDCL3 expression and immune infiltration. Finally, the results of this correlation analysis were visualized using the R package ggplot2.

### 2.7 Correlation analysis of PDCL3 with tumor mutation burden and microsatellite instability

Spearman’s correlation was used for the correlation analysis. The relationship between PDCL3 expression and TMB or MSI was analyzed separately. Radar plots were then applied to visualize the results of the correlation analysis.

### 2.8 DEGs and functional enrichment analysis

Tumor cases were divided into two groups based on the median PDCL3 expression level as the cut-off value. The R package limma was used to analyze the differences between the two groups, and the differentially expressed genes (DEGs) related to PDCL3 were identified. The threshold for filtering DEGs was set at an absolute log2 fold change ≥1.0. The DEGs were extracted, and the R package clusterProfiler was used to perform Gene Ontology (GO) and Kyoto Encyclopedia of Genes and Genomes (KEGG) analyses to explore the potential functional mechanisms of PDCL3 in tumors.

### 2.9 Studies of PDCL3 expression in cell clusters

The GSE189357, GSE242889, GSE263995, and GSE184198 datasets were used to analyze the expression levels of PDCL3 in cell clusters. The R package Seurat was used for quality control of all single-cell datasets. Dimensionality reduction and clustering were performed using uniform manifold approximation and projection (UMAP). Based on the marker genes of each cell cluster, the clusters were annotated using the CellMarker 2.0 database (http://bio-bigdata.hrbmu.edu.cn/CellMarker/).

### 2.10 Cell culture and RNA interference

The human NSCLC cell lines A549 and H1299 were obtained from the Chinese Academy of Sciences (Shanghai, China). Cells were cultured in Dulbecco’s Modified Eagle Medium (Gibco, Grand Island, USA) supplemented with 10% fetal bovine serum (FBS; Gibco) and 1% penicillin-streptomycin under standard conditions (37°C, 5% CO_2_). After 24 hours of incubation, siRNA transfection was performed using Lipofectamine 8000 (Beyotime Biotechnology, China) in serum-free medium. The siRNA sequences (designed and synthesized by Nanning Genesis Biotechnology Co., Ltd, China) were as follows:

- si-PDCL3-1: 5′-UGGAAUGACAUCUUACGCAAATT-3′,
- si-PDCL3-2: 5′-GCACCUUUACAAACAAGGAAUTT-3′,
- si-PDCL3-3: 5′-GCAUACCCAAUUAUCCUGAUATT-3′.

### 2.11 Real-time quantitative polymerase chain reaction

Total RNA was reverse-transcribed into cDNA using the PrimeScript RT Master Mix (Takara Bio, Japan). RT-qPCR was subsequently conducted on a LightCycler 480 system (Roche, Switzerland) with 10 μg of cDNA template, 2X Q3 SYBR qPCR Master Mix (ToloBio, China), and gene-specific primers. Gene expression levels were quantified using the 2-ΔΔCt method. The PDCL3 primer sequences (synthesized by Sangon Biotech, China) were as follows:

- Forward: 5′-GAATCTGCCCACGATATTTGTTTACC-3′,
- Reverse: 5′-TTCCATTCCAACTCATCTCTTGTCAG-3′.

### 2.12 Cell proliferation

Cell proliferation was assessed using the BeyoClick™ EdU-555 Cell Proliferation Detection Kit (Beyotime Biotechnology, China) and the Cell Counting Kit-8 (CCK-8; Beyotime Biotechnology). For EdU assays, cells were incubated with EdU solution 24 hours post-transfection. Nuclei were counterstained with Hoechst 33342, and fluorescence images were captured using an EVOS M7000 fluorescence microscope (Thermo Fisher Scientific, USA). For CCK-8 assays, 10% (v/v) CCK-8 reagent was added to the culture medium at the indicated time points (24, 48, and 72 hours). Absorbance was measured at 450 nm using a microplate reader (BioTek, USA).

### 2.13 Wound healing assay

Cells were seeded into six-well plates and cultured until reaching 80-90% confluency. A uniform wound was created in the monolayer using a sterile 200 μL pipette tip. After washing with PBS to remove detached cells, images of the wound area were acquired at 0 hours (baseline) and 24 hours post-wounding using a microscope (Nikon, Japan).

### 2.14 Transwell assay

Cell migration and invasion assays were performed using Transwell chambers (8 μm pore size; LABSELECT, China). Briefly, cells were harvested 24 hours post-transfection, resuspended in serum-free medium, and seeded into the upper chamber with 250 μL. The lower chamber was filled with 700 μL of complete medium containing 10% FBS. The chambers were incubated for 36 hours.

### 2.15 Statistical analysis

The t-test was applied to analyze the differential expression between two groups. Spearman’s correlation method was used for the correlation analysis. A p-value of less than 0.05 indicated that the results were statistically significant (ns, p > 0.05; *, p < 0.05; **, p < 0.01; ***, p < 0.001).

## 3 Results

### 3.1 Analysis of PDCL3 expression in pan-cancer

**Figure 1:**
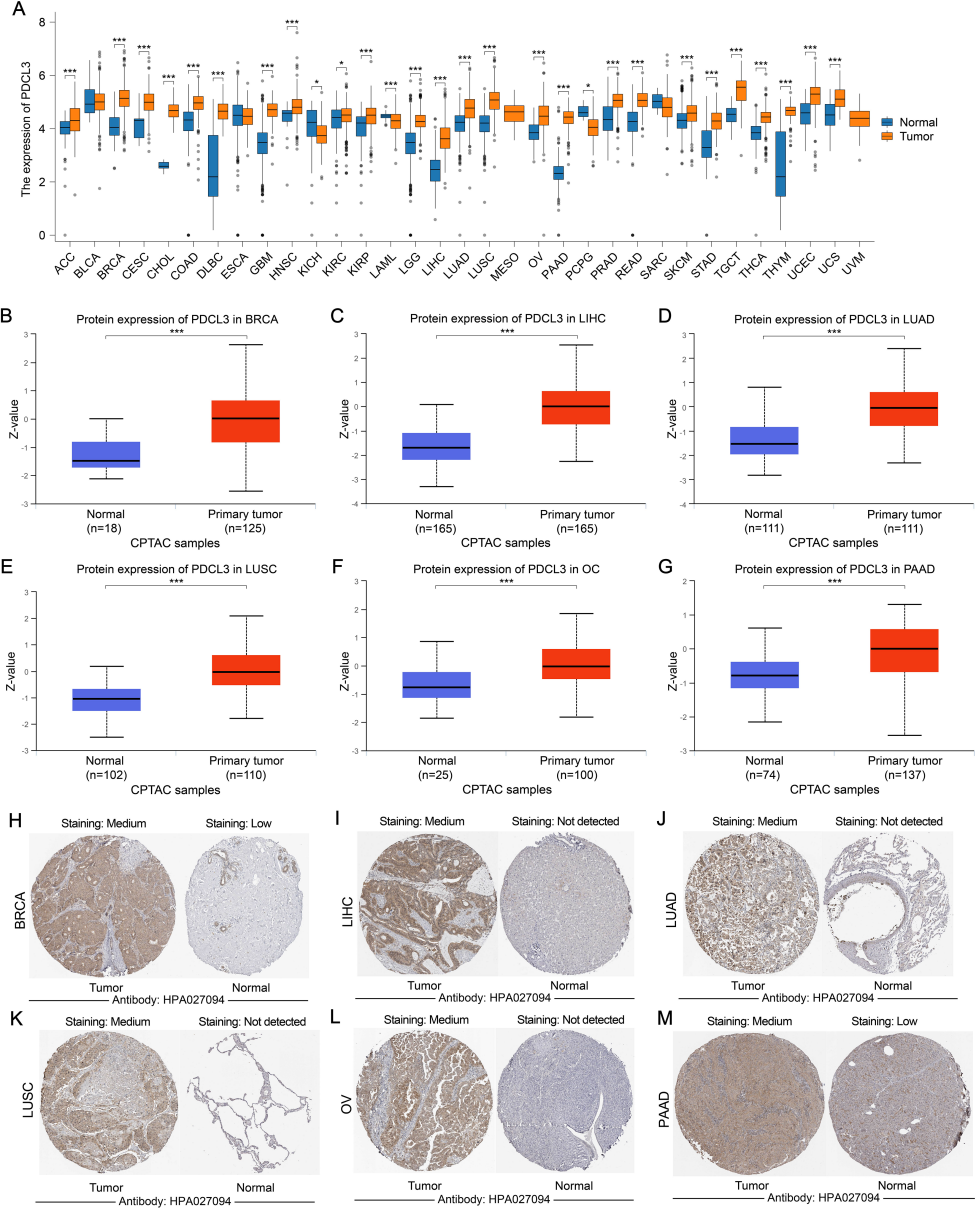
The expression of PDCL3 is upregulated in a variety of tumors. **(A)** Comparison of PDCL3 expression differences between tumor samples and corresponding paracancerous samples in various types of cancer. **(B–G)** Differential expression of PDCL3 from the UALCAN database between tumor and normal groups in BRCA, LIHC, LUAD, LUSC, OC, and PAAD). **(H–M)** Immunohistochemical images of PDCL3 in tumor and normal tissues, including BRCA, LIHC, LUAD, LUSC, OV, and PAAD, obtained from the HPA database. *p < 0.05; ***p < 0.001.

The pan-cancer analysis results indicated that PDCL3 was upregulated in most cancers (**Figure 1A**). Data from the UALCAN and HPA databases further confirmed that PDCL3 expression was upregulated in breast invasive carcinoma (BRCA), liver hepatocellular carcinoma (LIHC), lung adenocarcinoma (LUAD), lung squamous cell carcinoma (LUSC), ovarian cancer (OC), and pancreatic adenocarcinoma (PAAD) at the protein level (**Figures 1B–M**).

### 3.2 Diagnostic efficiency analysis of PDCL3 in pan-cancer

**Figure 2:**
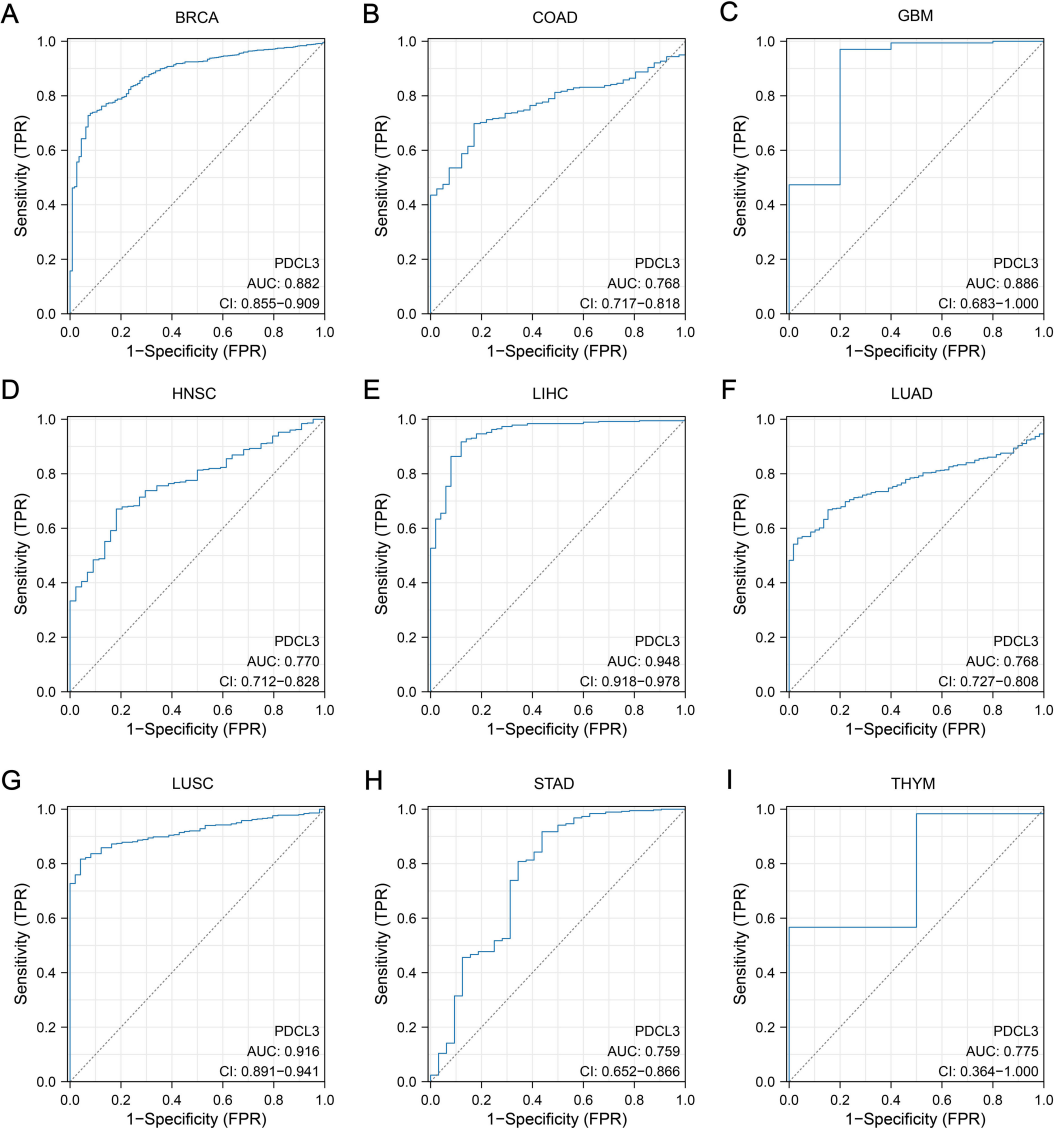
Diagnostic value of PDCL3. **(A)** Diagnostic ROC curves constructed based on PDCL3 expression levels in BRCA. **(B)** Diagnostic ROC curves constructed based on PDCL3 expression levels in COAD. **(C)** Diagnostic ROC curves constructed based on PDCL3 expression levels in GBM. **(D)** Diagnostic ROC curves constructed based on PDCL3 expression levels in HNSC. **(E)** Diagnostic ROC curves constructed based on PDCL3 expression levels in LIHC. **(F)** Diagnostic ROC curves constructed based on PDCL3 expression levels in LUAD. **(G)** Diagnostic ROC curves constructed based on PDCL3 expression levels in LUSC. **(H)** Diagnostic ROC curves constructed based on PDCL3 expression levels in STAD. **(I)** Diagnostic ROC curves constructed based on PDCL3 expression levels in THYM.

The area under the ROC curve (AUC) for PDCL3 in BRCA, COAD, GBM, HNSC, LIHC, LUAD, LUSC, STAD and THYM were 0.882, 0.768, 0.886, 0.770, 0.948, 0.768, 0.916, 0.759 and 0.775, respectively (**Figures 2A–I**). All AUC values were greater than 0.7, indicating that PDCL3 exhibited good diagnostic performance for these tumors.

### 3.3 Relationship between PDCL3 expression and tumor stage

**Figure 3:**
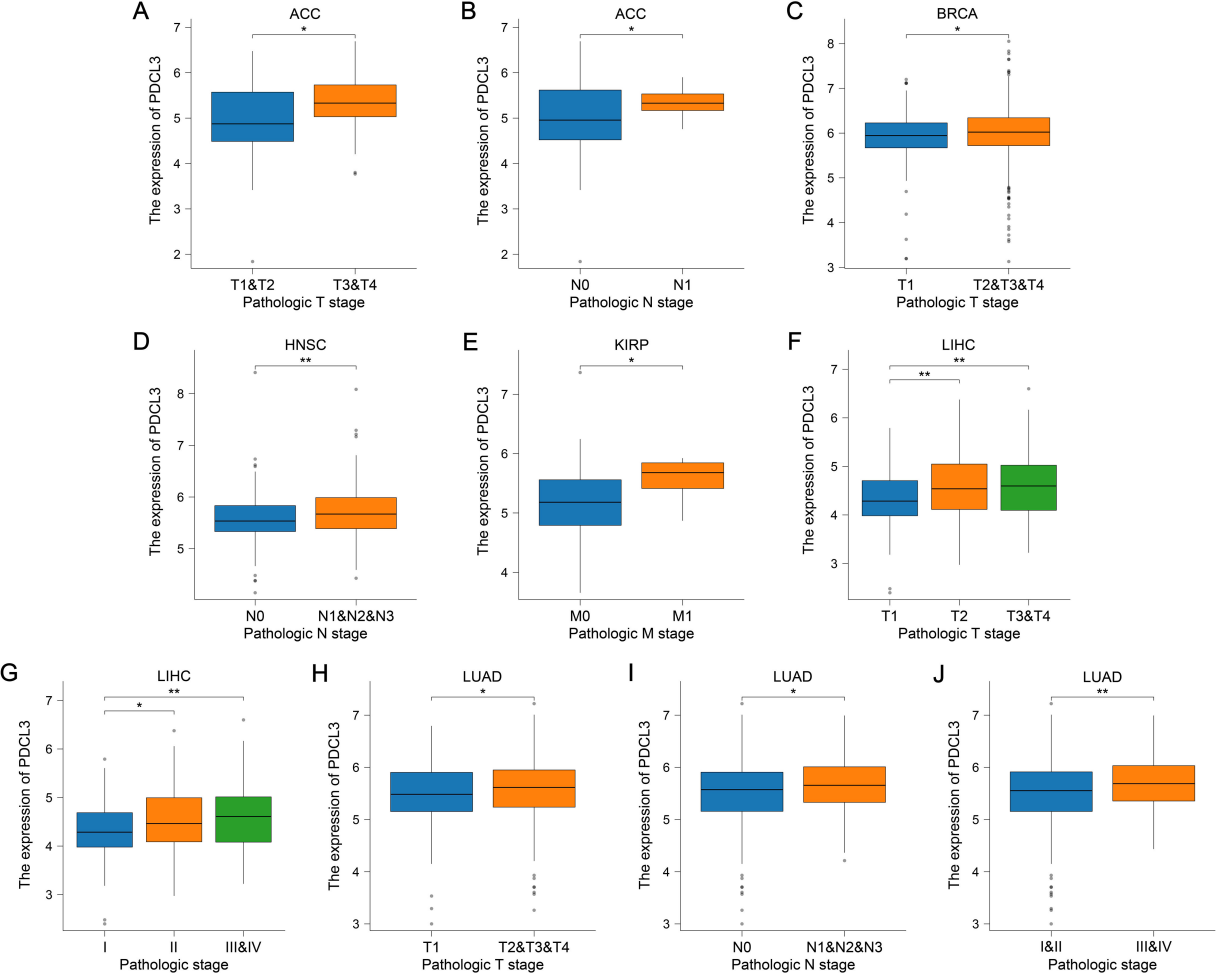
PDCL3 expression was upregulated in cases with advanced tumor stages. **(A, B)** Analysis of PDCL3 expression in ACC cases with different T stages or N stages. **(C)** Comparison of PDCL3 expression in BRCA cases with different T stages. **(D)** Comparison of PDCL3 expression in HNSC cases with different N stages. **(E)** Comparison of PDCL3 expression in KIRP cases with different M stages. **(F, G)** Differential analysis of PDCL3 expression in LIHC cases with different T stages or pathological stages. **(H–J)** Study of the relationship between PDCL3 expression and tumor stage in LUAD. *p < 0.05; **p < 0.01.

PDCL3 expression was upregulated in ACC, BRCA, LIHC, and LUAD samples with high T stage (**Figures 3A, C, F, H**). Additionally, PDCL3 tended to be highly expressed in ACC, HNSC, and LUAD samples with lymph node metastasis (**Figures 3B, D, I**), and was also highly expressed in KIRP cases with distant metastasis (**Figure 3E**). A significant difference in PDCL3 expression was observed among LIHC or LUAD samples across different pathological stages (**Figures 3G, J**).

### 3.4 KM survival analysis based on PDCL3 expression

**Figure 4:**
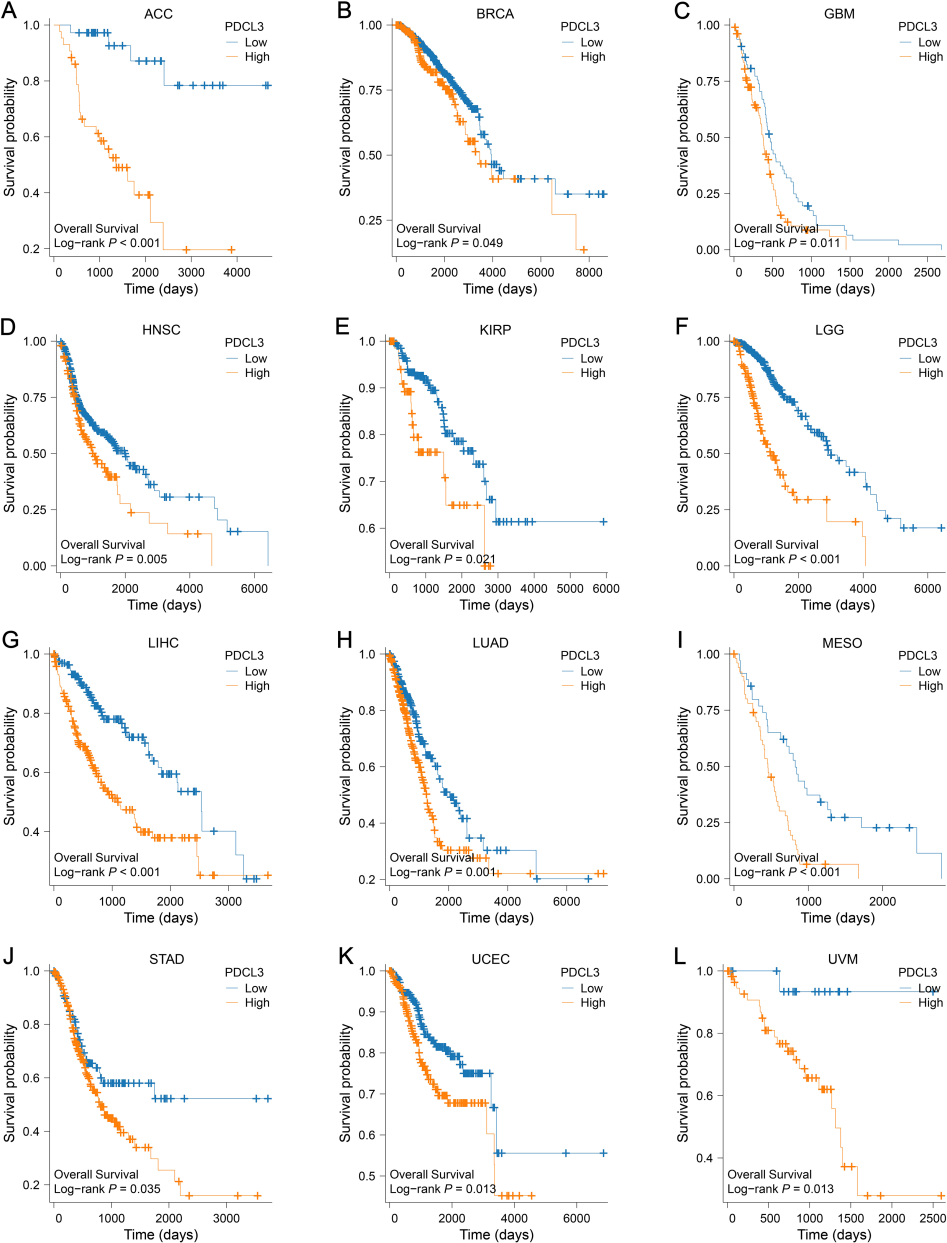
Prognostic value of PDCL3 in pan-cancer. **(A–L**) KM survival curve constructed by grouping according to the expression of PDCL3 in ACC, BRCA, GBM, HNSC, KIRP, LGG, LIHC, LUAD, MESO, STAD, UCEC, and UVM.

Survival analyses from the TCGA database revealed a worse outcome for tumor samples with upregulated PDCL3 expression (**Figures 4A–L**).

### 3.5 Analysis of genetic alterations in PDCL3

**Figure 5:**
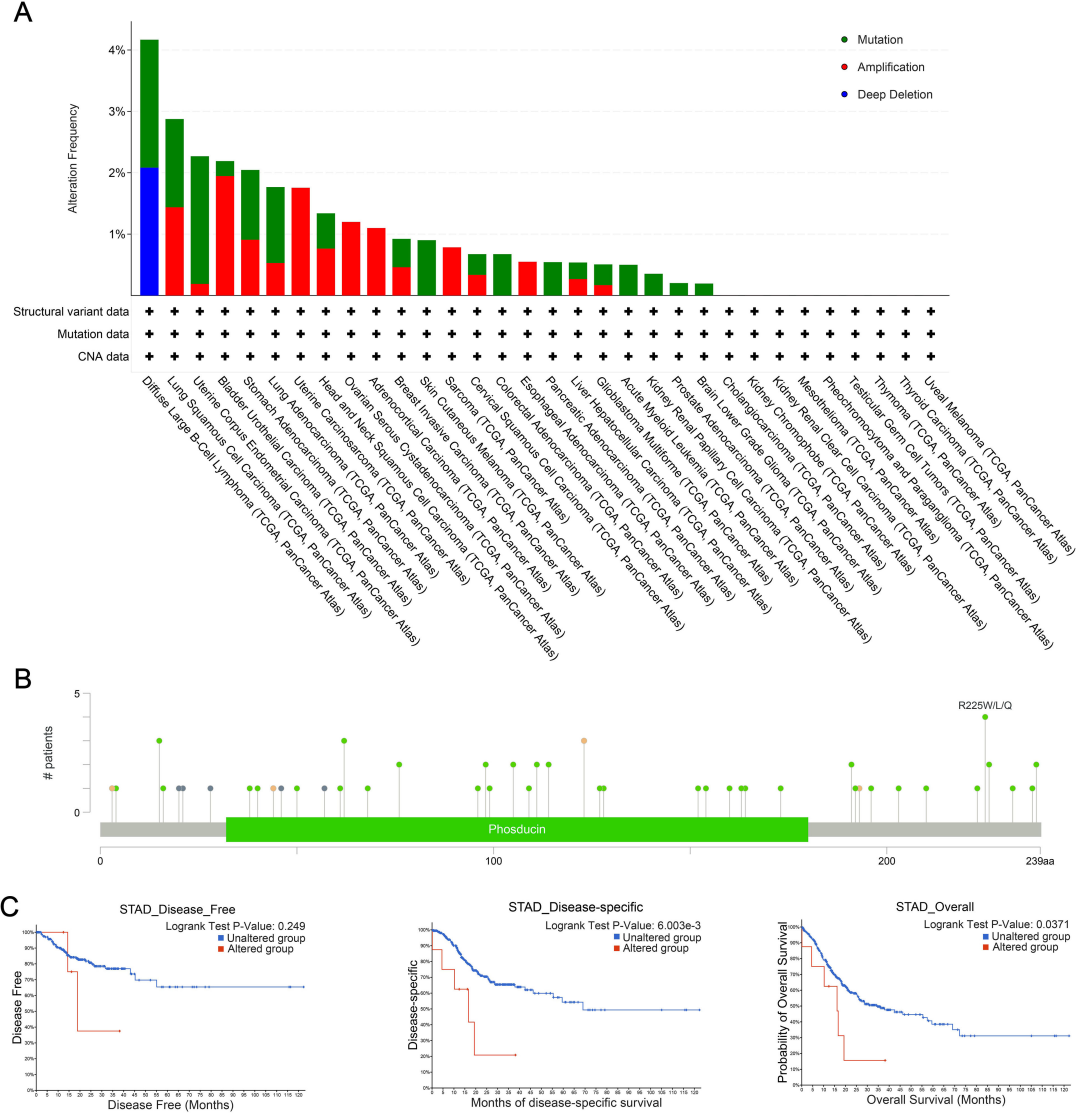
Genetic alteration properties of PDCL3 in pan-cancer. **(A)** Frequency and types of PDCL3 alterations in pan-cancer from TCGA. **(B)** Summary visualization of PDCL3 alterations from the cBioPortal database. **(C)** Potential relevance of alterations in PDCL3 to the prognosis of STAD.

The cBioPortal database was used to analyze genetic alterations of PDCL3 across different tumor types. Our analysis revealed that PDCL3 alterations in cancer were primarily mutations and amplifications (**Figure 5A**), with the most common alteration type being missense mutations (**Figure 5B**). Genetic alterations in PDCL3 were associated with poor overall survival and disease-specific survival in STAD, though no significant difference was observed in disease-free survival (**Figure 5C**).

### 3.6 Correlation analysis between PDCL3 expression and immune infiltration

**Figure 6:**
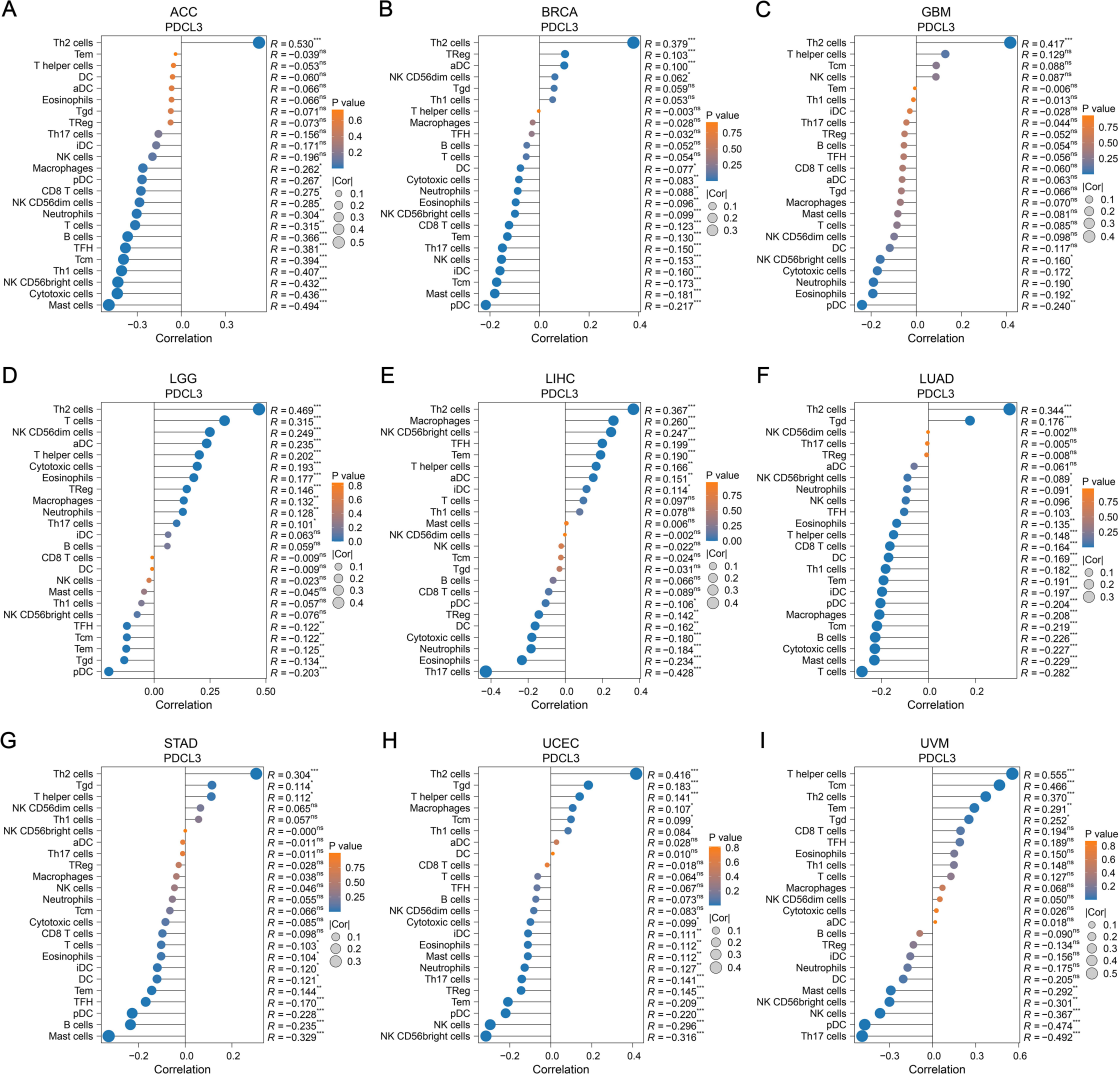
Relationship between PDCL3 and immune infiltration. **(A-I)** Study on the correlation between PDCL3 expression and immune infiltration in ACC, BRCA, GBM, LGG, LIHC, LUAD, STAD, UCEC, and UVM. In these dot plots, the size of the dot represents the correlation, and the color of the dot indicates the p-value. We set the absolute value of the correlation greater than 0.2 and a p-value less than 0.05 as the threshold.

In ACC, a positive correlation was observed between PDCL3 expression and T helper 2 (Th2) cell infiltration; In contrast, PDCL3 expression was negatively correlated with several immune cell types, including Mast cells, Cytotoxic cells, NK CD56bright cells, Th1 cells, Central memory T cells (Tcm), TFH, B cells, T cells, Neutrophils, NK CD56dim cells, CD8 T cells, plasmacytoid dendritic cells (pDCs), and Macrophages (**Figure 6A**). In BRCA and GBM, PDCL3 expression positively correlated with Th2 cell infiltration, but negatively correlated with pDC infiltration (**Figures 6B, C**). In LIHC,PDCL3 expression was positively associated with Th2 cells, Macrophages, and NK CD56bright cells, but negatively associated with Th17 cells and Eosinophils (**Figure 6E**). In LUAD, PDCL3 expression was positively correlated with Th2 cell infiltration, but inversely correlated with infiltration of seven other immune cell types, including T cells, Mast cells, Cytotoxic cells, B cells, Tcm, Macrophages, and pDC (**Figure 6F**). In STAD, PDCL3 expression showed a positive relationship with Th2 cells, but a negative correlation with Mast cells (**Figure 6G**). In uterine corpus endometrial carcinoma (UCEC), PDCL3 expression positively correlated with Th2 cells but was inversely correlated with NK CD56bright cells, NK cells, pDC, and Effector memory T cells (Tem) (**Figure 6H**). In uveal melanoma (UVM), PDCL3 expression was positively correlated with five immune cell types (Th cells, Tcm, Th2 cells, Tem, and T gamma delta cells [Tgd]) but negatively correlated with five others (Th17 cells, pDC, NK cells, NK CD56bright cells, and Mast cells) (**Figure 6I**). Taken together, the immune infiltration analysis indicates that PDCL3 expression consistently showed a positive correlation with Th2 cell infiltration across all tumor types, while it was inversely correlated with pDC infiltration, except in LIHC.

### 3.7 Association of PDCL3 with TMB and MSI

**Figure 7:**
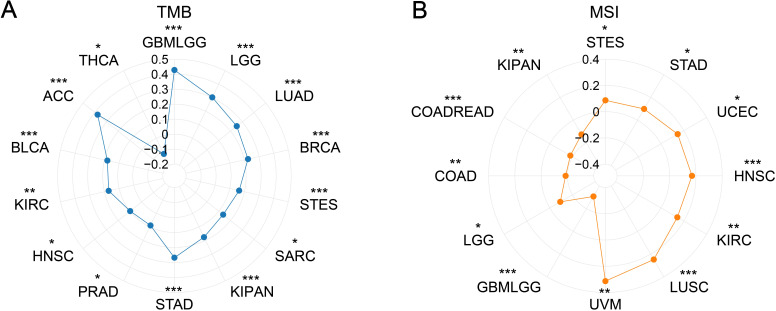
Correlation analysis between PDCL3 with TMB and MSI. **(A)** Investigation of the correlation between PDCL3 and TMB. **(B)** Investigation of the relationship between PDCL3 and MSI. *p < 0.05; **p < 0.01; ***p < 0.001.

PDCL3 expression showed a positive correlation with TMB in several cancers, including glioblastoma multiforme (GBMLGG, r =  0.4278), low grade glioma, LGG) (r  =  0.3031), LUAD (r  =  0.2533), BRCA (r  =  0.2250), stomach adenocarcinoma (STES, r  =  0.1644), sarcoma (SARC, r  =  0.1373), kidney pan-cancer (KIPAN, r  =  0.1762), stomach adenocarcinoma (STAD, r  =  0.2679), prostate adenocarcinoma (PRAD, r  =  0.0895), head and neck squamous cell carcinoma (HNSC, r  =  0.0992), kidney renal clear cell carcinoma (KIRC, r  =  0.1712), bladder urothelial carcinoma (BLCA, r  =  0.1802), and adrenocortical carcinoma (ACC, r  =  0.3764). In contrast, a negative correlation was observed between PDCL3 expression and TMB in thyroid carcinoma (THCA, r  =  -0.1158) (**Figure 7A**).

Regarding MSI, PDCL3 expression was positively correlated with MSI in STES (r  =  0.0867), STAD (r  =  0.0998), UCEC (r  =  0.1463), HNSC (r  =  0.1702), KIRC (r  =  0.1432), LUSC (r  =  0.2474), and UVM (r  =  0.3135). On the other hand, PDCL3 expression was negatively correlated with MSI in GBMLGG (r  =  -0.3073), LGG (r  =  -0.0920), COAD (r  =  -0.1861), colon adenocarcinoma and rectum adenocarcinoma (COADREAD, r  =  -0.1806), and KIPAN (r  =  -0.1246) (**Figure 7B**).

### 3.8 Functional enrichment analysis of PDCL3

**Figure 8:**
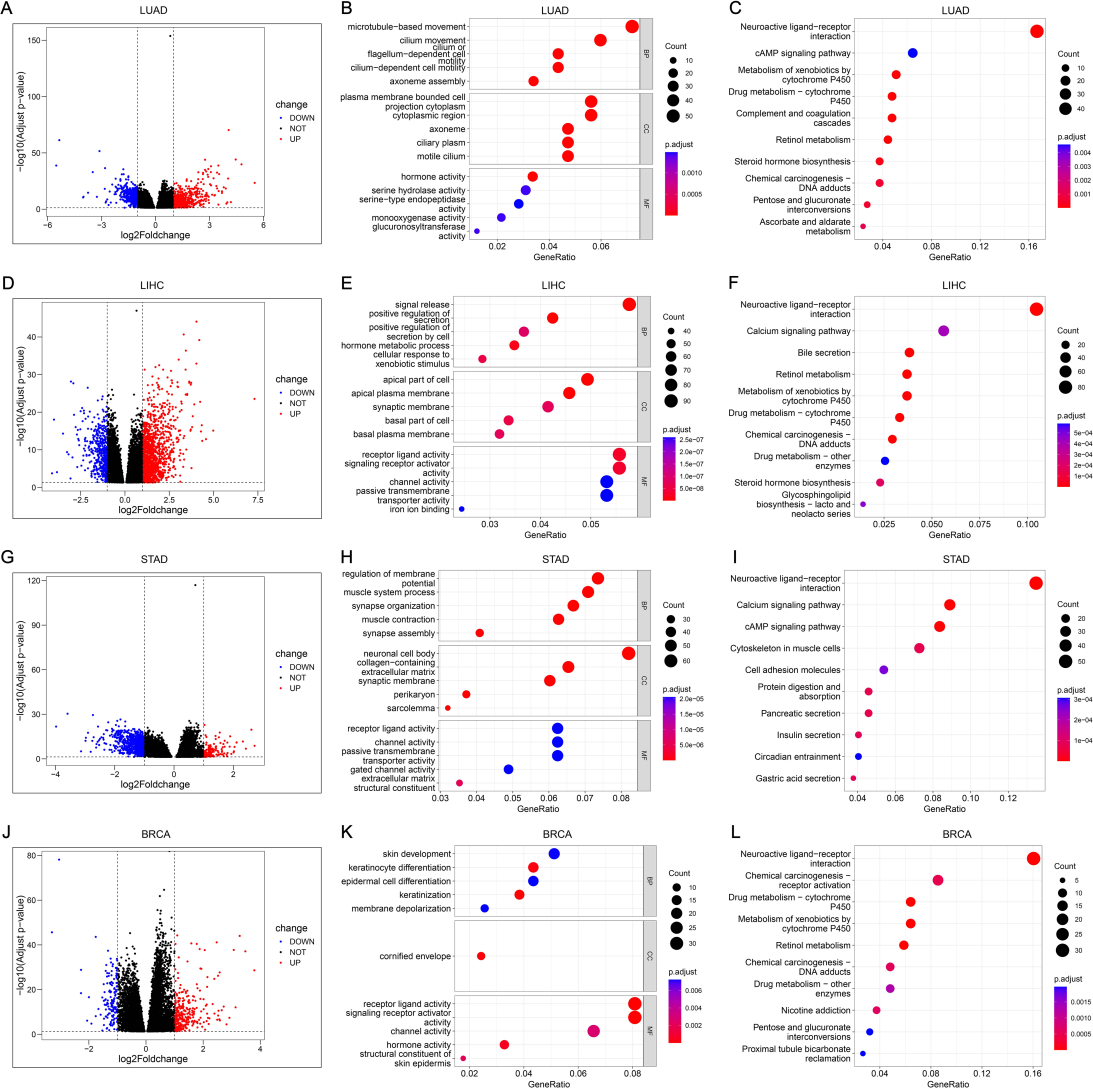
Functional analysis and annotation of PDCL3 in tumors. **(A)** Volcano map constructed from DEGs obtained by analysis of LUAD samples based on PDCL3 expression levels. **(B)** Dot plot demonstrating GO analysis of DEGs related to PDCL3 in LUAD. **(C)** Dot plot demonstrating KEGG analysis of DEGs related to PDCL3 in LUAD. **(D)** Volcano map constructed from DEGs obtained by analysis of LIHC samples based on PDCL3 expression levels. **(E)** Dot plot demonstrating GO analysis in LIHC. **(F)** Dot plot demonstrating KEGG analysis in LIHC. **(G)** Volcano map constructed from DEGs obtained by analysis of STAD cases based on PDCL3 expression levels. **(H)** Dot plot of the results obtained by performing GO analysis on DEGs in STAD. **(I)** Dot plot of the results obtained by performing KEGG analysis on DEGs in STAD. **(J)** Volcano map constructed from DEGs obtained by analysis of BRCA cases based on PDCL3 expression levels. **(K)** Demonstration of the results obtained from GO analysis of DEGs in BRCA by dot plot. **(L)** Demonstration of the results obtained from KEGG analysis of DEGs in BRCA by dot plot.

There were 917, 1810, 844, and 444 DEGs related to PDCL3 in LUAD, LIHC, STAD, and BRCA, respectively (**Figures 8A, D, G, J**). Functional enrichment analysis was conducted for the four groups of DEGs. The biological process (BP) was presented with 5 terms in GO analysis. DEGs of LUAD were concentrated in “microtubule-based movement”, “cilium movement”, “cilium or flagellum-dependent cell motility”, “cilium-dependent cell motility”, and “axoneme assembly” (**Figure 8B**). DEGs of LIHC were concentrated in “signal release”, “positive regulation of secretion”, “positive regulation of secretion by cell”, “hormone metabolic process”, and “cellular response to xenobiotic stimulus” (**Figure 8E**). DEGs of STAD were concentrated in “regulation of membrane potential”, “muscle system process”, “synapse organization”, “muscle contraction”, and “synapse assembly” (**Figure 8H**). DEGs of BRCA were concentrated in “skin development”, “keratinocyte differentiation”, “epidermal cell differentiation”, “keratinization”, and “membrane depolarization” (**Figure 8K**). Ten pathways annotated by DEGs were presented in KEGG analysis. DEGs of LUAD were clustered in the following terms: “Neuroactive ligand-receptor interaction”, “cAMP signaling pathway”, “Metabolism of xenobiotics by cytochrome P450”, “Drug metabolism-cytochrome P450”, “Complement and coagulation cascades”, “Retinol metabolism”, “Steroid hormone biosynthesis”, “Chemical carcinogenesis-DNA adducts”, “Pentose and glucuronate interconversions”, and “Ascorbate and aldarate metabolism” (**Figure 8C**). DEGs of LIHC were clustered in the following terms: “Neuroactive ligand-receptor interaction”, “Calcium signaling pathway”, “Bile secretion”, “Retinol metabolism”, “Metabolism of xenobiotics by cytochrome P450”, “Drug metabolism-cytochrome P450”, “Chemical carcinogenesis-DNA adducts”, “Drug metabolism-other enzymes”, “Steroid hormone biosynthesis”, and “Glycosphingolipid biosynthesis-lacto and neolacto series” (**Figure 8F**). DEGs of STAD were clustered in the following terms: “Neuroactive ligand-receptor interaction”, “Calcium signaling pathway”, “cAMP signaling pathway”, “Cytoskeleton in muscle cells”, “Cell adhesion molecules”, “Protein digestion and absorption”, “Pancreatic secretion”, “Insulin secretion”, “Circadian entrainment”, and “Gastric acid secretion” (**Figure 8I**). DEGs of BRCA were clustered in the following terms: “Neuroactive ligand-receptor interaction”, “Chemical carcinogenesis-receptor activation”, “Drug metabolism-cytochrome P450”, “Metabolism of xenobiotics by cytochrome P450”, “Retinol metabolism”, “Chemical carcinogenesis-DNA adducts”, “Drug metabolism-other enzymes”, “Nicotine addiction”, “Pentose and glucuronate interconversions”, and “Proximal tubule bicarbonate reclamation” (**Figure 8L**).

### 3.9 Analysis of PDCL3 expression in cell clusters of tumors

**Figure 9:**
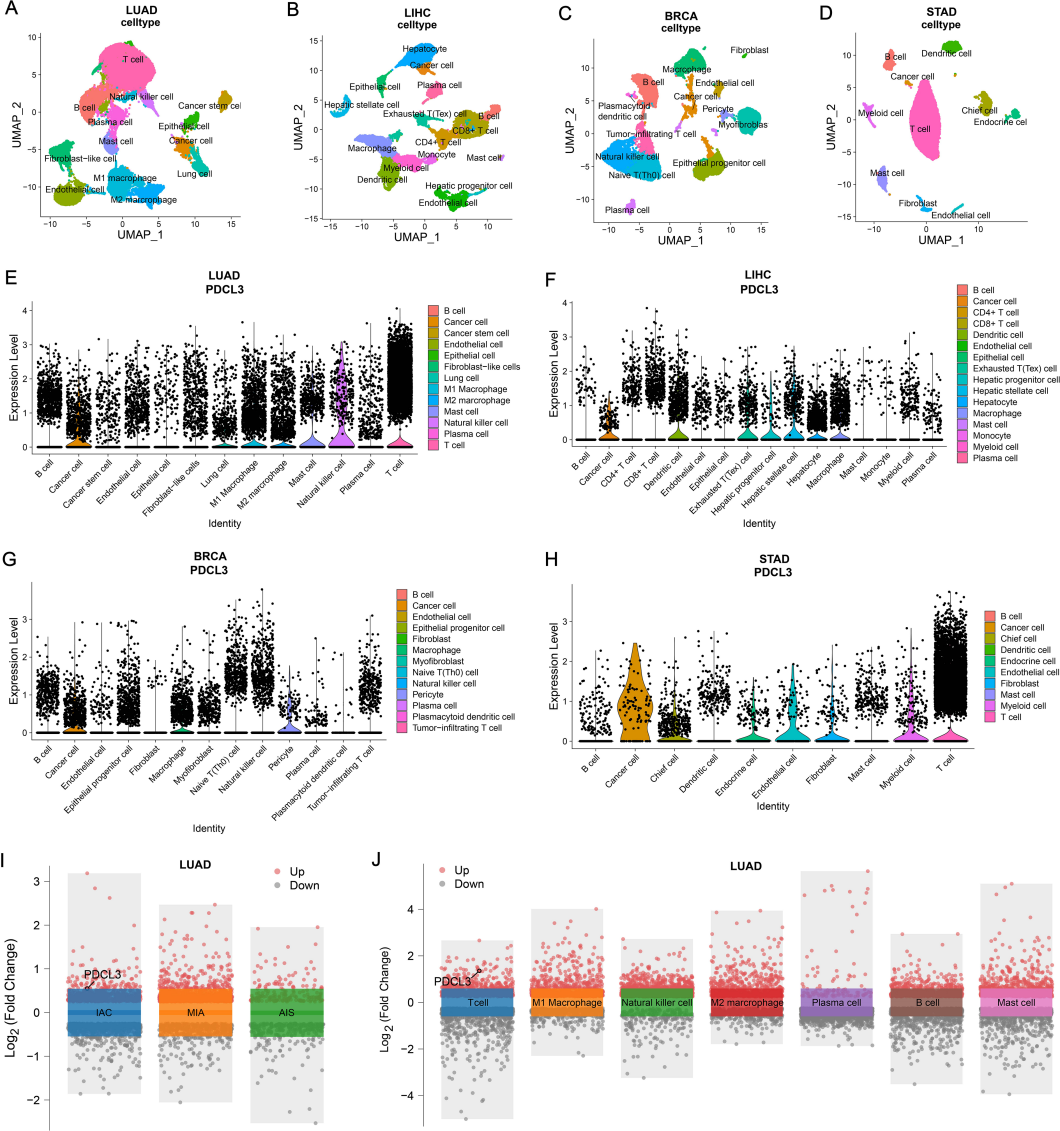
Comparison of PDCL3 expression at the cellular level. **(A)** UMAP plot for dimension reduction and cell annotation of LUAD single-cell sequencing data (GSE189357). **(B)** UMAP plot of dimension reduction and cell annotation in LIHC single-cell sequencing data (GSE242889). **(C)** UMAP plot presenting dimension reduction and cell annotation in BRCA single-cell sequencing data (GSE263995). **(D)** Demonstration of dimension reduction and cell clustering of STAD single-cell sequencing dataset (GSE184198) by UMAP plot. **(E)** Differential expression of PDCL3 at the cellular level in LUAD. **(F)** Expression analysis of PDCL3 at the single-cell sequencing level in LIHC. **(G)** Differences in PDCL3 expression at the BRCA single-cell sequencing level. **(H)** Comparison of PDCL3 expression between cell clusters in STAD. **(I)** Volcano plot of the differences between invasive adenocarcinoma (IAC), minimally invasive adenocarcinoma (MIA), and adenocarcinoma *in situ* (AIS) in the GSE189357 dataset. **(J)** Volcano plot of differential analysis between 13 sets of cell clusters in the GSE189357 dataset (this figure presents the results of differential analysis of immune cells only).

After dimensionality reduction and clustering of single-cell sequencing data, each cell cluster was annotated according to marker genes (**Figures 9A–D**). By analyzing the expression of PDCL3 in LUAD cell clusters, it was found that PDCL3 expression was mainly in cancer cells, lung cells, M1 macrophages, M2 macrophages, mast cells, natural killer cells, plasma cells, and T cells (**Figure 9E**). In LIHC, PDCL3 was predominantly expressed in cancer cells, dendritic cells, exhausted T (Tex) cells, hepatic progenitor cells, hepatic stellate cells, hepatocytes, and macrophages (**Figure 9F**). Analysis of PDCL3 expression in BRCA cell clusters revealed that PDCL3 expression was primarily in cancer cells, macrophages, and pericytes (**Figure 9G**). In STAD, the expression of PDCL3 was most prominent in cancer cells, chief cells, endocrine cells, endothelial cells, fibroblasts, myeloid cells, and T cells (**Figure 9H**). In the differential analysis of single-cell data from LUAD, PDCL3 was upregulated in IAC compared to MIA and AIS, suggesting that PDCL3 may promote the invasive growth of LUAD (**Figure 9I**). Differential analysis was performed in 13 cell clusters from LUAD, and PDCL3 was upregulated in T cells compared to other cell clusters, indicating that the role of PDCL3 in LUAD may be related to T cells (**Figure 9J**).

### 3.10 Functional verification of PDCL3 in NSCLC cells

**Figure 10:**
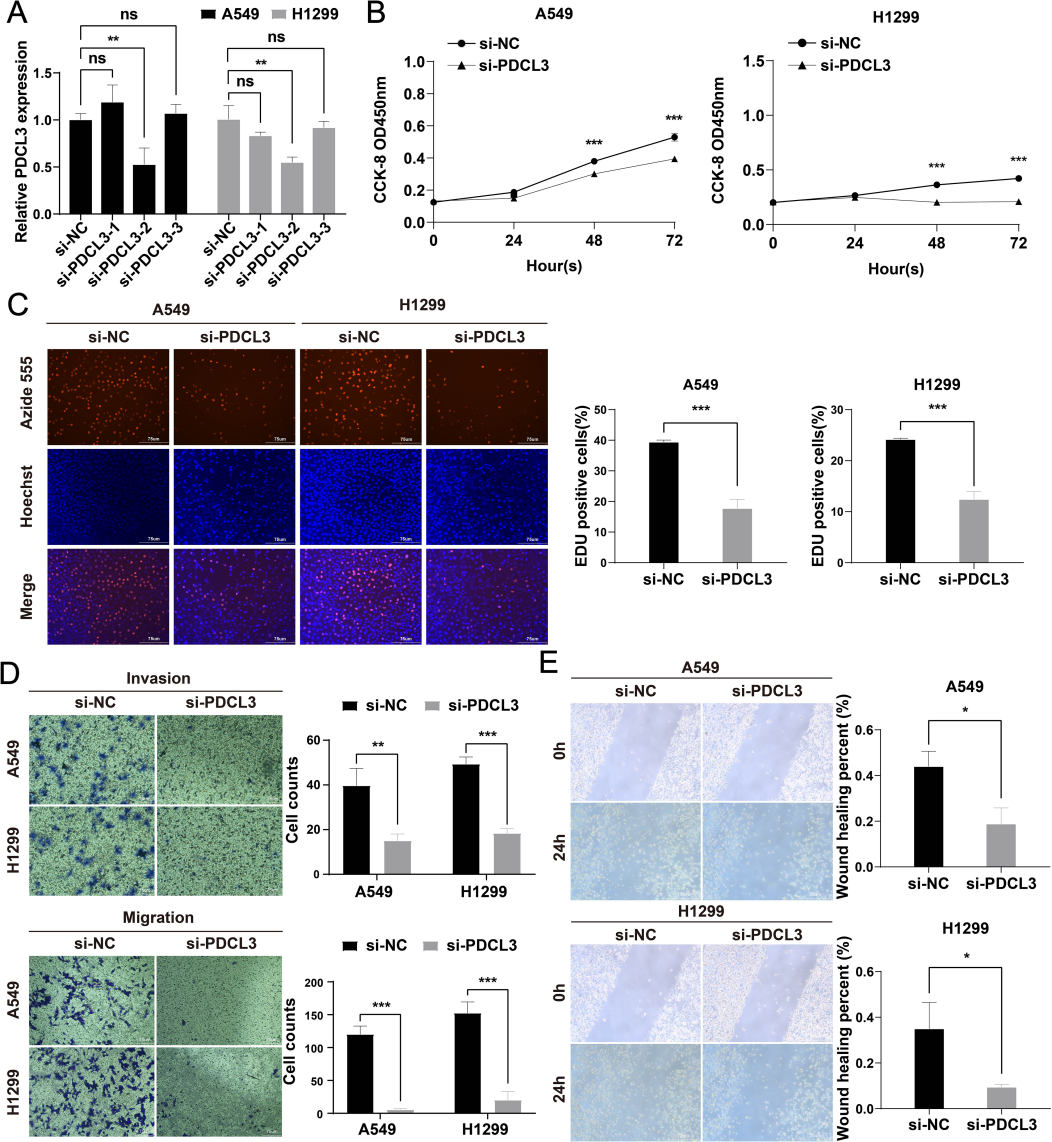
Functional characterization of PDCL3 in NSCLC progression. **(A)** PDCL3 mRNA expression in A549 and H1299 cells post-transfection with siRNA (si-PDCL3-2: targeting sequences). **(B)** CCK-8 viability curves showing time-dependent proliferation suppression in PDCL3-depleted cells. **(C)** EdU incorporation assay quantifying DNA replication rates. Red: EdU-positive nuclei; Blue: Hoechst 33342 counterstain. **(D)** Transwell migration and Matrigel-based invasion assays. **(E)** Scratch wound closure kinetics monitored at 0/24 h Migration distance calculated as percentage wound closure. *p < 0.05; **p < 0.01; ***p < 0.001.

To investigate the functional role of PDCL3 in NSCLC, si-PDCL3 was applied to A549 and H1299 cell lines. RT-qPCR analysis confirmed a significant reduction in PDCL3 mRNA levels following transfection with si-PDCL3-2, validating the silencing efficiency (**Figure 10A**). Functional assays were subsequently performed to assess the phenotypic consequences of PDCL3 depletion. CCK-8 and EdU proliferation assays revealed that PDCL3 knockdown significantly suppressed NSCLC cell growth, as evidenced by decreased absorbance values and EdU-positive cell ratios (**Figures 10B, C**). Furthermore, Transwell migration/invasion assays demonstrated that PDCL3 silencing attenuated both the migratory and invasive capacities of NSCLC cells (**Figure 10D**), and impaired migration was observed in the scratch wound closure assay (**Figure 10E**).

## 4 Discussion

Initial studies revealed the presence of PDCL3 in the retina and pineal gland, confirming that it mainly functions in the visual system and is regarded as a photoreceptor (10). In recent years, PDCL3 has been found to be localized in the cytoplasm and endoplasmic reticulum, with its expression linked to the promotion of angiogenesis, primarily through its involvement in regulating VEGFR-2 expression (8, 11). Angiogenesis is a critical process in tumor metastasis, driven by VEGFR-2, and PDCL3 can bind to VEGFR-2 to inhibit its degradation and ubiquitination, thereby regulating tumor progression (12). As a hallmark of cancer development and progression, abnormal angiogenesis is a complex process regulated by both anti-angiogenic and pro-angiogenic factors (13). Anti-angiogenic therapy targeting angiogenesis-related factors is considered a promising treatment for malignant tumors (14, 15). The promotive role of PDCL3 in hepatocellular carcinoma and glioma has been reported previously, and its diagnostic and prognostic value has been confirmed in these two diseases (16–18). The expression of PDCL3 in colorectal cancer was also extracted to construct a prognostic model, which effectively predicted the overall prognosis of patients (19). This study aimed to comprehensively analyze the expression, diagnostic and prognostic value, immune regulation, gene mutation, and potential function of PDCL3 in pan-cancer, revealing its role in cancers at the single-cell transcriptome level.

In our results, we confirmed that PDCL3 was overexpressed in nearly all types of malignant tumors in the TCGA dataset. The upregulation of PDCL3 in BRCA, LIHC, LUAD, LUSC, OC, and PAAD was further confirmed at the protein expression level using data from the CPTAC and HPA databases. These findings support the consistency of PDCL3 expression at both the mRNA and protein levels across multiple cancers. By analyzing differential gene expression, especially at the protein level, biomarkers with diagnostic and prognostic value for tumors can be identified (20, 21). The diagnostic and prognostic value of PDCL3 was evaluated using diagnostic ROC curves and KM survival analysis. The results confirmed that PDCL3 had high efficiency in distinguishing tumors from normal samples in BRCA, COAD, GBM, HNSC, LIHC, LUAD, LUSC, STAD, and THYM, with upregulation of PDCL3 expression correlating with poor prognosis in ACC, BRCA, GBM, HNSC, KIRP, LGG, LIHC, LUAD, MESO, STAD, UCEC, and UVM. Additionally, mutations in PDCL3 were associated with poor outcomes in STAD. Subsequently, we assessed the relationship between PDCL3 and tumor stage, and the results indicated that PDCL3 expression was correlated with T stage in ACC, BRCA, LIHC, and LUAD, lymph node metastasis in ACC, HNSC, and LUAD, and distant metastasis in KIRP. In the analysis of single-cell datasets, PDCL3 expression was detected at high levels in LUAD, LIHC, BRCA, and STAD cancer cells, with PDCL3 upregulated in IAC, suggesting that PDCL3 may promote the invasive growth of LUAD. In addition, cell function experiments confirmed that PDCL3 could enhance the proliferation, migration, and invasion of cancer cells. These findings underscore the significance of PDCL3 in the development of malignant tumors.

TME plays a critical role in tumor growth, metastasis, and resistance to chemotherapy and immunotherapy. It is a highly complex system composed of tumor cells, immune cells, stromal cells, and endothelial cells (22–25). This study primarily explored the relationship between PDCL3 and the tumor microenvironment, focusing on immune infiltration. We found that PDCL3 was positively correlated with Th2 cell infiltration but negatively correlated with pDC infiltration. Additionally, single-cell sequencing analysis revealed that PDCL3 was upregulated in T cells. Th cells contain two major subsets, Th1 and Th2, and the imbalance between these subsets is a decisive factor in the progression of malignant tumors (26). Existing studies have shown that Th2 cells are associated with the progression and metastasis of cancer, exerting a pro-tumor effect (27, 28). However, they have also been confirmed to have an anti-tumor effect, which mainly depends on the type and stage of the tumor (29, 30). T cells are closely linked to pro-angiogenesis, primarily through the secretion of heparin-binding epidermal growth factor and pro-angiogenic factors such as fibroblast growth factor 2 (31). In the TME, pDCs are considered negative regulators of T cell responses, and their activation can induce the progression of anti-tumor immunity (32).Within the TME, CD8+ T cells are critical components of anti-tumor immunity, functioning as primary effector cells with indispensable therapeutic potential (33, 34). We found an inverse correlation between PDCL3 expression and CD8+ T cell infiltration in ACC, BRCA, and LUAD, suggesting the potential role of PDCL3 in modulating anti-tumor immunity. To further explore the potential of PDCL3 in tumor immunotherapy, we investigated its relationship with TMB and MSI. TMB is defined as the number of mutations per megabase of DNA in a cancer (35), while MSI results from defects in the mismatch repair system (36). Both TMB and MSI are valuable biomarkers for predicting a patient’s response to immunotherapy (37). Our findings suggest that PDCL3 is related to immune infiltration and immunotherapy response. Therefore, PDCL3 could serve as a potential marker for predicting immunotherapy outcomes and may regulate the immune microenvironment of tumors.

To investigate the potential function of PDCL3 in cancer, four types of cancers (LUAD, LIHC, STAD, and BRCA) were selected as representatives for enrichment analyses. KEGG results revealed that DEGs related to PDCL3 in LUAD and STAD were enriched in the cAMP signaling pathway, while DEGs related to PDCL3 in LIHC and STAD were enriched in the calcium signaling pathway. The cAMP signaling pathway influences the biological functions of malignant cells primarily by controlling cell signaling and regulating the transcription of PKA and CREB. When the cAMP/PKA/CREB pathway is activated and interacts with other signaling pathways, it can contribute to the development of malignant tumors. Studies have shown that the expression and activation of CREB are linked to tumor growth, and PKA is considered a potential marker for tumor identification and treatment (38). Recent studies have confirmed that the cAMP signaling pathway can upregulate several oncogenes, although it can also inhibit some oncogenes. Its effect on tumors is context-dependent, varying according to the type of cancer and the tumor microenvironment (39, 40). For example, activation of the cAMP signaling pathway promotes tumor progression in leukemia, lung cancer, gastric cancer, liver cancer, and prostate cancer (41–48). In contrast, in diffuse large B cell lymphoma, medulloblastoma, and basal cell carcinoma of the skin, cAMP signaling has an inhibitory effect on tumor development (49–51). Dysregulation of calcium signaling is also implicated in tumorigenesis, especially when the disruption of calcium-mediated cell death mechanisms leads to pathological conditions. This highlights the critical role of the calcium signaling pathway in tumor progression (52, 53). In conclusion, the functional enrichment results suggest that PDCL3 may play a role in tumor progression through both the cAMP and calcium signaling pathways.

In summary, our study explored the potential of PDCL3 as a diagnostic and prognostic marker in tumors, highlighted its role in immune regulation, and provided new insights into cancer progression and immunotherapy research. However, there are some limitations that should be acknowledged. Primarily, the analysis was based on bioinformatics data from public databases, which may have an insufficient sample size, potentially leading to errors. Additionally, the direct mechanism of PDCL3 in cancer has not yet been confirmed, and further experimental studies are needed to better understand its precise role.

## 5 Conclusions

PDCL3 was demonstrated to be a potential marker for pan-cancer diagnosis and immunotherapy, and its high expression was closely related to the poor prognosis in cancer patients. In LUAD, the cancer-promoting mechanism of PDCL3 may involve the induction of angiogenesis through the regulation of T cell secretion functions.

## Data Availability

The original contributions presented in the study are included in the article/supplementary material. Further inquiries can be directed to the corresponding authors.
